# Gastric Carcinoid and Obesity: Association or Coincidence? Report of Two Cases and Literature Review

**DOI:** 10.1155/2013/848075

**Published:** 2013-01-15

**Authors:** Obaid Al-Harbi, Mustafa Shakir, Nabeel Al-Brahim

**Affiliations:** ^1^Department of Surgery, Farwaniya Hospital, 81004 Sabah Al-Naser, Kuwait; ^2^Department of Pathology, Farwaniya Hospital, P.O. Box 3313, 22034 Salmiya, Kuwait

## Abstract

Bariatric surgery is a prevalent procedure due to the high incidence of obesity and comorbidities. Upper gastrointestinal endoscopy is one of the procedures used to evaluate the patient before surgery. However, its role is questionable. The incidental findings during endoscopy are variable including inflammatory diseases, and ulcers, and epithelial and stromal tumors. Herein a report of two obese sisters with incidental gastric carcinoids was diagnosed in prebariatric surgery endoscopy. *Case Summary*. 35- and 41-year-old female patients presented with obesity and BMI of 102 and 46 kg/m^2^, respectively. Both patients underwent upper gastrointestinal endoscopy as part of presurgical evaluation. Multiple polyps were indentified in both patients, and biopsy was taken. Histological examination revealed tumors that were formed by nests of epithelial cells. The cells have eosinophilic cytoplasm and monomorphic nuclei, typical morphology of neuroendocrine tumors. *Conclusions*. (1) Upper gastrointestinal endoscopy is an important procedure for prebariatric surgery evaluation. (2) Gastric carcinoid is a rare tumor with higher incidence among obese patients.

## 1. Introduction

Laparoscopic sleeve gastrectomy is a procedure that became more prevalent as a treatment for morbid obesity due to marked improvement in surgical techniques. However, patients going for this procedure need proper preoperative assessment including medical, laboratory, and psychological evaluation [1]. Upper gastrointestinal endoscopy (UGIE) is one of the investigations that should be considered for prebariatric surgery evaluation. But its role is still questionable. Incidental pathological findings during prebariatric surgery evaluation are many and variable including epithelial and stromal tumors, peptic ulcers, and inflammatory conditions. Humphreys et al. reported abnormalities by prebariatric UGIE in 56% of their series of patients, two of them were esophageal adenocarcinoma [2]. Muñoz and his colleagues [3] were able to demonstrate different abnormalities in their 626 series of patients including duodenal and gastric ulcers, polyps, Barrette's esophagus, and gastric cancer.

Gastric carcinoid tumor is one of the rare tumors that were discovered during prebariatric UGIE. In a series of 426 patients who underwent bariatric operation, carcinoid tumor was diagnosed preoperatively in only one patient [4]. Similarly, there were additional four cases reported in the literature [5–7]. In this paper another two cases of such tumor are reported in two sisters going for sleeve gastrectomy with a review of the previous cases. A discussion about the relation of obesity and carcinoid tumor will be touched.

## 2. Case Report

### 2.1. Case 1

**Figure 1:**
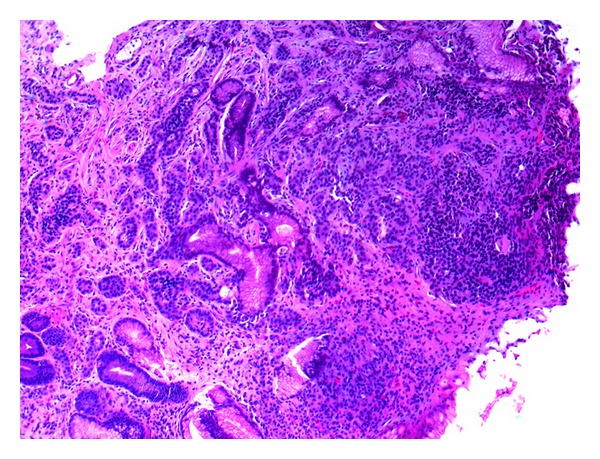
Histological morphology of gastric carcinoid consists of well-differentiated nests with granular cytoplasm and round to oval nuclei (H&E).

**Figure 2:**
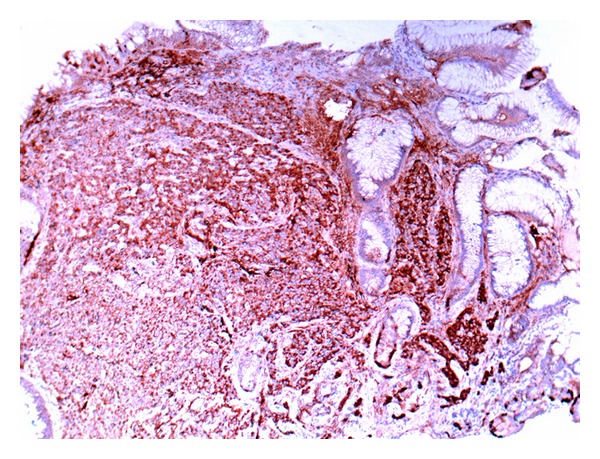
Immunohistochemical stains demonstrate the expression of chromogranin, a neuroendocrine marker.

 A 35-year-old female with body mass index (BMI) of weight 40.8 kg/m² with no history of any comorbidity underwent UGIE as a workup prior to laparoscopic sleeve gastrectomy (LSG). Endoscopy revealed a gastric body polyp that measured 3.0 × 2.0 cm with central ulceration. Two smaller satellite polyps were seen near the main lesion, and biopsy was taken. Histopathological examination of all lesions was the same and revealed tumor cells that were arranged in small nests with abundant granular cytoplasm and round to oval nuclei (Figure 1). The tumor cells were positive for cytokeratin and neuroendocrine markers including chromogranin, synaptophysin, and NSE, diagnostic of gastric carcinoid (Figure 2). Ultrasound was done routinely prior to bariatric surgery and showed a cyst in the right lobe of the liver. Eventually, a computed tomography (CT) scan of abdomen was performed which confirmed the presence of multiple rounded lesions in the greater curvature of the stomach. In addition, there was a solitary, subcapsular mass in segment 8 of the liver measuring 1.8 cm × 1.3 cm × 1.6 cm suggestive of metastasis. A CT-guided liver biopsy targeting the mass was done which showed hepatocytes with preserved architecture and foci of steatosis. Malignancy was not seen. Following the diagnosis of gastric carcinoid with a possible liver metastasis, the plan was changed to laparoscopic total gastrectomy with lymphadenectomy, Roux-en-Y gastrojejunostomy, and simultaneous biopsy of the liver mass. Postoperatively she did well and no leaks on gastrografin swallowed that was performed on the third postoperative day.

Histopathology of liver lesion again showed preserved architecture with microsteatosis and no tumor deposit. The gastric specimen showed well-differentiated neuroendocrine tumor with lymphatic permeation. The surrounding gastric tissue shows variable degree of G-cell hyperplasia. There were tumor deposits in the serosa, and two out of nine lymph nodes showed metastasis with perinodal fat infiltration. 

Postoperative octreotide scan showed features in keeping with liver metastasis. MRI was performed and revealed a small lesion 1.7 × 1.4 cm in segment 6 of the liver suggestive of metastasis. Another focal lesion in segment 8 was noted, which was peripherally located that measured 2.1 × 1.9 cm probably site of previous biopsy. Based on imaging suspicion of liver metastasis, the patient underwent segmental liver resection in the cancer center, and histopathological examination proofed the presence of metastatic neuroendocrine tumor in the liver.

### 2.2. Case 2

Three months later, her elder sister, 41 year old with BMI 46 m²/kg, was subjected to UGIE as preoperative assessment for sleeve gastrectomy. The UGIE showed multiple tiny nodules and sessile polyps at the gastric body and antrum. Histopathological examination revealed tumor nests with typical morphology of well-differentiated neuroendocrine tumor that expressed chromogranin and synaptophysin. Subsequently, the patient underwent CT scan of the abdomen and chest that uncovered incidental multiple small polyps in the sigmoid colon about 6 mm in diameter. Colonoscopy was done, and the polyps were biopsied which did not reveal any significant pathological changes. At this point the patients decided to think of surgical intervention due to her psychological stress. Two months later, the patient underwent an UGIE which revealed no gross abnormality. Therefore, the patient decided to undergo sleeve gastrectomy. The specimen received demonstrated granular mucosa with no definite tumor nodules. Histological examination revealed multiple foci of microscopic neuroendocrine tumor with the largest focus that measured 0.4 cm in a background of G-cell hyperplasia. Both patients were investigated for possibility of other associated tumors and were negative.

## 3. Discussion

As communities go into more civilization and change thier the life style, the population pays taxes of increasing morbidities including diabetes, hypertension, and obesity. As a result, bariatric surgery becomes more prevalent especially with the presence of advanced surgical techniques. Patients going for such procedures need special evaluation. One of the investigations commonly performed for such patients is UGIE. However, the significance of such procedure is debatable and depends on the significance of the findings that can be diagnosed by such procedure. Schirmer and his colleagues [8] published one of the largest series of patients underwent UGIE as preparation for bariatric surgery. Twenty-six patients out of 536 (4.9%) patients had significant abnormalities that need to change the operative procedure including: grade 2 esophagitis, gastric or duodenal ulcer, hiatal hernia and gastric polyps. In another more recent study [6], the authors found that the prevalence of clinically significant findings was 12%. The findings included were Barrett's esophagus, gastric ulcers, duodenal ulcers, and gastric carcinoid. On the contrary to previous two studies with a relatively low prevalence of endoscopic findings, there were studies with high percentage of clinical findings. A study by Madan et al. [9] uncovered that the percentage of patients with clinical findings who underwent UGIE was 90%; almost all of them were hiatal hernias. Furthermore, a study [10] showed that the percentage of findings in preoperative UGIE was 67%. These findings include hiatal hernias, esophagitis, peptic ulcer disease, gastric polyps, and early-stage esophageal carcinoma. This variability in the findings is unclear; however, some authors claim that the selection criteria for patients going for UGIE may play a role as the symptomatic patients are more prone to get abnormal findings. 

Gastric carcinoid tumor is a rare epithelial neoplasm of the stomach. It comprises less than 1% of the all gastric neoplasms. Gastric carcinoids are subclassified into three types: those associated with atrophic gastritis and pernicious anemia, those associated with Zollinger-Ellison syndrome with multiple endocrine neoplasia (MEN I), and sporadic cases. The tumors associated with atrophic gastritis are the most common and accounted for 70–80% of gastric carcinoids [11]. Type 1 and type 2 gastric carcinoids are commonly small, multiple, and located in the mucosa and submucosa. Also, they are usually associated with less lymph node metastasis. On the other hand, type 3 carcinoid is commonly single, larger, and associated more with deeper invasion and more lymph node metastasis [11]. Incidental gastric carcinoids diagnosed by prebariatric surgery UGIE are rare with only five cases that have been reported in the literature [4–7]. By looking to the previously reported cases of gastric carcinoid in prebariatric patients (Table 1), we can extrapolate some of the following findings: all cases were reported in female patients with age ranged from 32 to 41. The majority of patients had BMI ranging between 40 and 46. Five out of 6 patients had type 1 carcinoid tumor, with typical endoscopic appearance of type 1, as small multiple polyps. However, one patient had type 3 carcinoid tumor that is sporadic with classic morphology as a large tumor measured 6.0 cm. 

Carcinoid tumor is a rare tumor in general population with incidence variable between 1 and 2.5 per 100,000 population. However, an analysis of large series of cases of such tumors demonstrated increasing the incidence of carcinoid tumors in all sites particularly gastric and rectal carcinoids [12]. There were no clear reasons for the increase, but the authors attributed this to more awareness of this tumor and improvement in diagnostic technology. In the study performed by Mottin et al. [7], they found that the incidence of carcinoid tumor in their series of obese patients was 0.358% that is equal to (358 per 100,000). This incidence is higher than previously mentioned general population incidence. Therefore, they believed that there was some association between carcinoid tumors and obesity. Similarly, in our series two of gastric carcinoids were diagnosed out of 863 (0.23%) cases of obese patients who underwent bariatric surgery. This makes the overall incidence 230 per 100,000 population more than the general population and similar to the finding previously reported by Mottin et al. [7]. This observation further supports the hypothesis that carcinoid tumor has an association with obesity. Mottin and his colleagues did not have an explanation for this association. However, they suggested that the metabolic effects of obesity can be related to this higher incidence. Interestingly, if we go back to see all that cases of gastric carcinoid that have been reported in obese patients (Table 1), we will see that 5 out 6 cases were type 1 gastric carcinoid. Type 1 has well-known association with gastric atrophy and G-cell hyperplasia, the precursor cells for carcinoid tumors. In animal study [13], Campos et al. proved that obese Zuker rat had antral G-cell hyperplasia in comparison to lean animals. Also, they proved that dietary restriction to these obese animals will reduce antral G-cell hyperplasia comparable to lean animals. Therefore, they concluded that G-cell hyperplasia was a result of hyperphagia and abnormal feeding behavior. From these observations of previously reported cases and animal study we can extrapolate that these gastric carcinoids are probably related to abnormal feeding behavior of the obese patients.

In conclusion, prebariatric surgery upper gastrointestinal endoscopy is an important tool to evaluate the patients as abnormal pathology can be diagnosed and will change the management plan. Carcinoid tumor is rare in general population; however, observations showed higher incidence in obese patients. There is no definite explanation to this association. But the observation of previously reported cases and animal study raised the possibility of abnormal dietary behavior, and hyperphagia has a role in the association. Therefore, we need more studies on human to see these associations. 

## Figures and Tables

**Table 1:** Summary of previously reported cases of gastric carcinoid diagnosed by pre-briatric surgery UGIE.

Case	Sex	Age	BMI	Carcinoid type	Endoscopic findings
Case 1 [4]	NA	NA	NA	Type 1	small polyp
Case 2 [5]	F	32	44.4	Type 1	multiple polyps
Case 3 [6]	NA	NA	NA	NA	NA
Case 4 [7]	F	41	45.2	Type 3	Solitary tumor
Case 5 [7]	F	32	44.4	Type 1	Multiple polyps
Case 6*	F	35	102	Type 1	Multiple polyps
Case 7*	F	41	46	Type 1	Multiple polyps

*Current cases.
